# Antibacterial Food Packaging with Chitosan and Cellulose Blends for Food Preservation

**DOI:** 10.3390/polym17131850

**Published:** 2025-07-02

**Authors:** Tengfei Qu, Xiaowen Wang, Fengchun Zhang

**Affiliations:** 1 Xinjiang Key Laboratory of Clean Conversion and High Value Utilization of Biomass Resources, College of Chemistry and Chemical Engineering, Yili Normal University, Yining 835000, China; 2 Sichuan Water Development Group Co., Ltd., Chengdu 610000, China; wxwlx3041461@163.com; 3 School of Materials and Environmental Engineering, Chengdu Technological University, Chengdu 611730, China

**Keywords:** food packaging, chitosan, cellulose, food preservation

## Abstract

With the increasing demand for food quality and the need for green and sustainable development of food packaging materials in the environment, the preparation and optimization of multifunctional natural and renewable antibacterial packaging materials have become an important trend. This article aims to explore the development of chitosan–cellulose composite materials with good antibacterial properties and promote the widespread application of chitosan and cellulose in food packaging materials. Combining various natural polysaccharide polymers, we discuss the application of chitosan cellulose in meat, dairy products, fruits and vegetables, and fishery products. Meanwhile, we explore their antibacterial and antioxidant behaviors during their use as food packaging materials. This provides a reference for effectively improving the performance of modified chitosan and cellulose food packaging materials in the future. Based on the above explanation, we analyzed the advantages and disadvantages of modified chitosan and cellulose and looked forward to the future development trends of chitosan and cellulose blend films in food preservation. Chitosan–cellulose blends not only have important prospects in food packaging and preservation applications, but can also be combined with intelligent manufacturing to enhance their food preservation performance. The aim of this review is to provide valuable references for basic research on the antimicrobial properties of these composites and their practical application in smart food packaging.

## 1. Introduction

Food spoilage is an important cause of food safety, as food becomes inedible due to spoilage [1]. Food spoilage leads to significant food waste and economic losses. About 1.3 billion tons of grain are wasted annually globally, and food companies lose USD 70 billion annually [2]. This waste also consumes other resources used in the food production process, such as water, energy, and labor [3]. At the same time, new opportunities and demands have emerged in the emerging market for fresh fruit and vegetable products. This demand stems from applying innovative packaging solutions and preservation technologies to ensure food quality and freshness [4,5]. Different antibacterial properties in food packaging are crucial for enhancing food safety and extending shelf life [6]. Antibacterial packaging is an effective measure to resist microbial contamination and foodborne diseases and to maintain the freshness of food by preventing the proliferation of these microorganisms [7]. Furthermore, innovative antibacterial packaging solutions can gain a market advantage [8]. This method can also reduce the dependence of food on chemical preservatives. In short, new food packaging is crucial for safeguarding public health and promoting the development of high-quality food products.

Traditional food packaging materials include plastics (e.g., polyethylene, polypropylene), glass, and metal. They have long been used to protect food from spoilage by providing barriers to moisture, oxygen, light, and contaminants [9,10,11]. However, these materials come with some limitations. Primarily, traditional plastics lack inherent antimicrobial properties. They are rarely able to inhibit the growth of bacteria, molds, and pathogens [3,12]. Secondly, many of these materials are derived from non-renewable resources. It is contributing to significant environmental pollution. The worst thing is that if applied improperly, certain synthetic food packaging materials may leach harmful substances that may pose potential risks to food health. Additionally, glass has several drawbacks, including brittleness, heavy weight, and high processing costs. Metals also have their own disadvantages, such as high costs and difficulties in recycling and disposal.

In contrast, some antibacterial food packaging is made from chitosan and cellulose blends. They offer a more effective and sustainable solution for food preservation [13]. Chitosan possesses natural antimicrobial properties. Combining it with cellulose produces a packaging material that actively inhibits microbial growth [14]. These biopolymers are biodegradable and derived from renewable resources. They are an environmentally friendly alternative to traditional materials. Moreover, chitosan–cellulose blends can be engineered to improve mechanical strength and barrier properties. These properties contain moisture and gas permeability more effectively than conventional packaging [15]. This innovation reduces environmental impact and meets food packaging demand.

**Figure 1:**
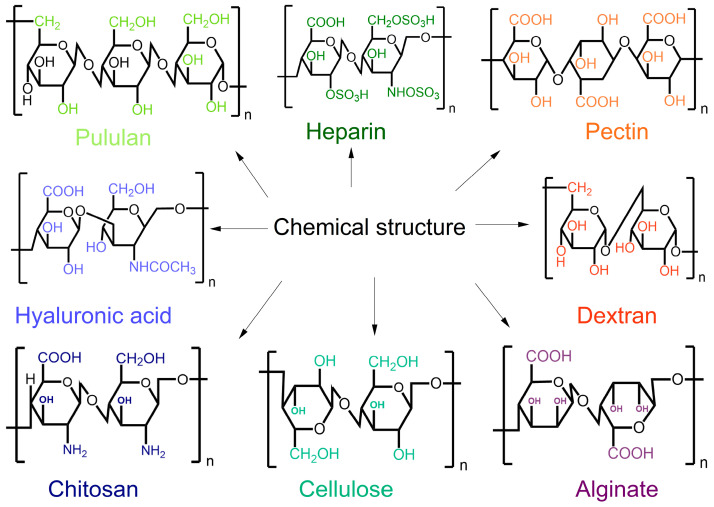
The chemical structures of different natural polysaccharides.

Chitosan and cellulose are natural polysaccharides. Their structures and chemical compositions vary depending on their sources. Common natural polysaccharides include cellulose, chitosan, lignin, and so on, as shown in Figure 1. These polysaccharides are typically formed by different monosaccharide units linked together by glycosidic bonds. Chitosan and cellulose play important roles in various applications across multiple industries [16]. Chitosan is derived from chitin, a polysaccharide found in the exoskeletons of crustaceans and certain fungi. Through deacetylation, chitin is converted into chitosan, resulting in a biopolymer that exhibits excellent biocompatibility, biodegradability, and antimicrobial properties [17,18]. These characteristics make chitosan particularly valuable in biomedical applications, food preservation, and agriculture [19]. Cellulose is the most abundant biopolymer on Earth. It is primarily present in the cell walls of plants, algae, and some bacteria [20]. Composed of long chains of β-D-glucose units linked by β-1,4-glycosidic bonds, cellulose provides structural strength to plant cells [21,22]. It is known for its exceptional tensile strength, hydrophilicity, and biodegradability. Due to these properties, cellulose is extensively used in textiles, paper, and packaging material [23,24].

Both chitosan and cellulose are increasingly being explored for their potential in innovative applications [25]. Their natural origins and favorable properties position them as key players in biodegradable materials [26]. Chitosan exhibits good antibacterial properties; however, its mechanical performance is suboptimal, which is improved through blending with cellulose. Chitosan is distributed within the cellulose matrix, forming a composite structure. The presence of cellulose not only serves as a carrier for chitosan but also facilitates the controlled release of the antimicrobial agent—chitosan [19,21,27]. Researchers continue to enhance these materials’ functionality through chemical modifications. So, it is beneficial for a more sustainable future of antibacterial food packaging.

Concern over food safety, quality, and sustainability is becoming increasingly prominent. It is urgent to explore innovative packaging solutions. This review aims to provide insight into the role of chitosan and cellulose blends in antibacterial food packaging materials, to enhance food preservation. First, we explore their chemical and physical properties, elucidating their contributions to antimicrobial activity and packaging performance. Secondly, this review details the mechanisms of their microbial growth inhibition, including their effects on microbial cell membranes. Furthermore, this review analyzes current methods for preparing chitosan–cellulose blends and discusses their different properties, advantages, and drawbacks. Case studies highlighting the application of these materials in various food products have been included. Finally, this review addresses the environmental impact and application potential of chitosan and cellulose in food packaging. Overall, this review seeks to provide an integrated understanding of the antibacterial properties of chitosan and cellulose blends, fostering further research and development in food packaging.

## 2. Applications and Preparations of Chitosan–Cellulose Packaging in Food Preservation

Fresh meat, dairy products, fruits, and vegetables are prone to spoilage, often due to microbial contamination. Therefore, developing effective food packaging strategies is crucial. The combination of chitosan and cellulose has shown promising potential in antimicrobial food packaging, as it offers a new solution for extending the shelf life of food and enhancing safety. Fresh produce, particularly delicate items like berries, cut fruits, and leafy greens, has a short shelf life and is prone to spoilage. Utilizing packaging made from chitosan–cellulose mixtures can significantly improve its freshness and nutritional quality. This material provides effective moisture and toxin barriers that inhibit the growth of spoilage microorganisms, thereby reducing food waste.

Due to differences in their characteristics, meat and poultry products have varied preservation requirements. Chicken, a high-protein and perishable fresh ingredient, is particularly susceptible to microbial contamination during storage. Therefore, strict control of microbial growth is essential to maintain its freshness [27]. In contrast, beef features coarser fibers and varied fat distribution, necessitating preservation methods that inhibit surface microbial proliferation and prevent the deterioration of meat quality due to oxidation and other factors [28].

Dairy products are rich in moisture and nutrients, making them highly susceptible to microbial contamination and rapid oxidation [29]. Their preservation must focus on the dual needs of inhibiting microbial growth and preventing oxidation. Therefore, it is essential to choose packaging materials that effectively suppress microbes, provide good sealing performance, and are opaque to block light, which can lead to nutrient degradation.

Bread, when fresh, has a soft and tender texture; however, its high moisture content makes it particularly vulnerable to drying out and mold growth [30]. The preservation of bread must address the need to inhibit microbial growth and prevent oxidation while also maintaining moisture levels. Thus, it is important to select packaging materials that can fulfill these requirements and possess a certain level of compressive strength to protect the bread from damage during transport and storage.

Sausages, as processed meat products, have complex compositions containing various spices, additives, and so forth. Their preservation must address the dual challenge of inhibiting microbial growth and preventing the oxidation and rancidity of fats [31]. Cooked foods, having undergone preparation, are more easily exploited by microorganisms, and their flavor compounds are prone to volatilization [32]. Thus, besides antibacterial action, preservation of these products requires efforts to maintain flavor and texture.

The advanced antibacterial packaging technology made from chitosan and cellulose specifically addresses these diverse needs [33,34,35]. For chicken, it significantly reduces surface microbial loads, minimizing spoilage risks. For beef, it suppresses bacteria while slowing down oxidation processes. In the case of sausages, it effectively prevents fat oxidation and inhibits bacterial growth. For cooked foods, this packaging minimizes flavor loss, ensuring food safety while maximizing the preservation of the texture, quality, and unique flavors of various products, ultimately enhancing preservation effectiveness and consumer experience. This type of packaging is particularly vital in retail environments, where the risk of contamination can be higher due to extended display times [34,35]. Table 1 showcases specific cases of chitosan and cellulose blends applied in preserving fresh meat, dairy products, fruits, and vegetables, clearly delineating their potential in food packaging.

In addition to fresh produce and meats, the dairy industry can reap significant benefits from using chitosan–cellulose blends in food packaging. Products such as cheese, yogurt, and milk are susceptible to microbial spoilage, affecting their taste, texture, and safety. The incorporation of biodegradable and antibacterial packaging can extend the shelf life of these items by controlling microbial growth and moisture loss. For instance, soft cheeses and yogurts, which have higher moisture content, are especially prone to spoilage, and chitosan’s antimicrobial properties can help preserve their quality throughout the storage period [59].

Bakery products represent another category where chitosan and cellulose blends can improve packaging effectiveness. Bread and pastries are common victims of mold growth and staleness due to moisture absorption [60]. Antibacterial packaging, which uses chitosan to inhibit mold formation while regulating moisture levels, can help maintain the freshness and palatability of baked goods. This is particularly beneficial for artisanal breads and goods with no preservatives, ensuring that consumers receive products that are not only safe but also have desirable textures and flavors when consumed.

Seafood is another highly perishable category that stands to benefit significantly from chitosan–cellulose packaging. Fish and shellfish are particularly prone to spoilage and bacterial growth, necessitating careful preservation methods. Antibacterial packaging can help extend the shelf life of seafood by minimizing microbial contamination, thus ensuring quality and safety. Whether dealing with fresh fish or ready-to-eat seafood salads, the use of these bio-based packaging materials can significantly enhance the product’s longevity without compromising flavor or texture [61]. Finally, the blending of chitosan and cellulose for antibacterial food packaging presents a multi-faceted solution that can enhance the safety, quality, and shelf life of a wide range of food products, from fresh produce and meats to dairy, bakery, seafood, and ready-to-eat meals. This innovation not only aids in reducing food waste but also aligns with the growing consumer demand for sustainable and health-conscious packaging solutions.

## 3. Antibacterial Action and Antioxidant Properties

### 3.1. Interaction of Cellulose Blends with Microbials

**Figure 2:**
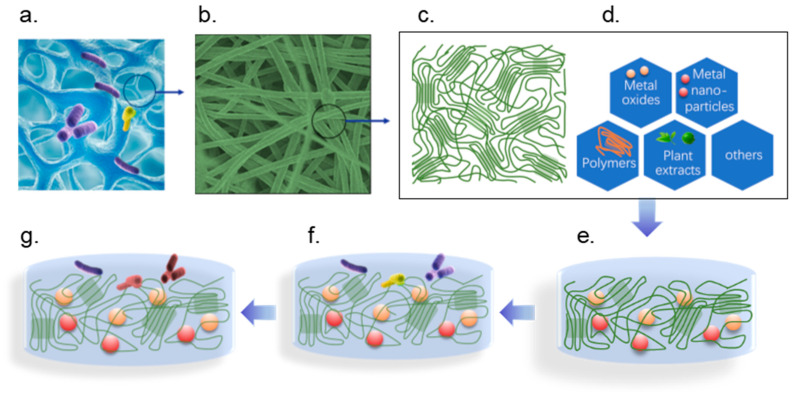
The schematic of the interaction of cellulose blends with microbials. (**a**) The surface of the cellulose with microbials; (**b**,**c**) the microstructure of the cellulose is composed of the crystalline fraction and the amorphous fraction; (**d**) the cellulose composite with different antibacterial active substances; (**e**–**g**) the antibacterial process of the cellulose composite with different antibacterial active substances. (Green thread represents cellulose, purple and yellow cylinders represent active bacteria, and reddish-brown cylinders represent dead bacteria.)

Cellulose is a natural polysaccharide primarily found in plant cell walls and does not possess antimicrobial activity. However, after chemical modification or when combined with other active components, it may acquire certain antimicrobial functions. Cellulose blends exhibit unique interactions with microbials in food preservation [13]. These blends create a physical barrier that can prevent microbial access to food surfaces, reducing the likelihood of contamination. The high surface area of cellulose fibers facilitates the adsorption of antimicrobial compounds released from chitosan. It promotes a synergistic effect that actively disrupts microbial growth. This interaction can significantly lower the initial microbial load on food products, improving safety and extending shelf life. The synergistic effects of cellulose with other natural compounds, such as essential oils, metal oxides, or plant extracts, were explored. The schematic of the interaction of cellulose blends with microbials is shown in Figure 2 [62]. These potential applications of cellulose in food preservation are expected to expand further [63].

Once microbial cells interact with cellulose–chitosan blends, several mechanisms can cause cell damage or death. Chitosan’s cationic nature allows it to interact electrostatically with negatively charged microbial cell membranes and the cell membranes in a destabilized state [64]. This disruption increases the permeability of the cell, causing essential intracellular components to leak out and ultimately leading to cell lysis. Additionally, binding chitosan to the cell surface can interfere with microbial metabolic processes, hindering their growth and reproduction. This dual action—physical barrier and chemical interaction—makes cellulose blends particularly effective against pathogens, including bacteria and fungi.

Moreover, adding cellulose blends into food packaging offers the potential for sustained release of active compounds. This slow-release mechanism ensures that antimicrobial activity is maintained over an extended period. It provides continuous protection against microbial contamination. The biocompatibility and non-toxicity of cellulose and its blends make them suitable for contact with food because they do not pose risks of harmful chemical migration. Ultimately, the interaction of cellulose blends with microbial cells represents a promising approach to developing effective, safe, and environmentally friendly food packaging solutions.

### 3.2. Chitosan Antibacterial Activity

**Figure 3:**
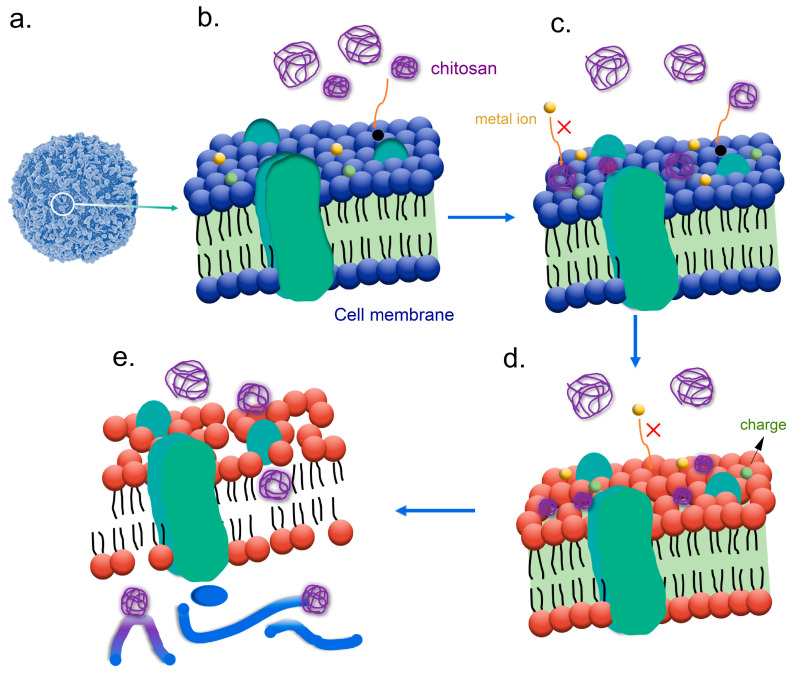
Schematic mechanisms of the antibacterial activity of chitosan. (**a**) A schematic of a microbial cell. (**b**) Microbial cell membranes. (**c**–**e**) The different-molecular-weight chitosan interacts with the microbial cell membranes and the internal DNA fractions.

Chitosan is a natural biopolymer derived from chitin [14]. The effectiveness of chitosan as an antibacterial agent can be primarily attributed to its chemical structure. The interactions of chitosan with the microbial cell (Figure 3a) membranes are shown in Figure 3b. Chitosan is a cationic polymer with positive charges. It plays a pivotal role in its antibacterial mechanism. When the positively charged chitosan is introduced to bacterial cells, it interacts with the negatively charged components of the bacterial cell membrane, destroying the membrane integrity as shown in Figure 3c,d. This disruption can result in increased permeability and ultimately leakage of essential intracellular components, leading to cell death. High-molecular-weight chitosan typically has a molecular weight exceeding 100,000 Mw, making it relatively difficult to dissolve in neutral pH environments. In contrast, low-molecular-weight chitosan usually has a molecular weight below 50,000 Mw, which allows for better solubility and a wider range of applications. A blend of high- and low-molecular-weight chitosan components demonstrates robust antifungal activity. The high-molecular-weight components disrupt the stability of fungal membranes, facilitating the penetration of low-molecular-weight components into the cells, where they interfere with vital cellular processes [64,65,66,67].

In addition to membrane disruption, chitosan can affect microbial physiology by interfering with cellular processes. Once chitosan penetrates the bacterial cell wall, it can inhibit vital metabolic functions by interacting with intracellular proteins and nucleic acids. This interaction not only halts protein synthesis but may also impair DNA replication, which is critical for the survival and proliferation of bacteria, as shown in Figure 3e. Consequently, the broad-spectrum antibacterial activity of chitosan makes it effective against various microbes, including both Gram-positive and Gram-negative bacteria. Its ability to inhibit the growth of pathogenic bacteria, such as Escherichia coli and Staphylococcus aureus, highlights its potential applicability in food preservation and packaging [64,68].

Additionally, the antibacterial efficacy of chitosan can be affected by various factors, such as its molecular weight, degree of deacetylation, and concentration in formulations. Generally, chitosan with a lower molecular weight demonstrates greater effectiveness against bacteria. This enhanced performance is attributed to its improved solubility and greater capacity to interact with microbial cells. The degree of deacetylation, which refers to the extent to which chitin has been converted to chitosan, affects the polymer’s charge density and, consequently, its antibacterial activity. Studies have shown that chitosan with a higher degree of deacetylation exhibits enhanced antibacterial properties. Furthermore, variations in pH and ionic strength can also impact chitosan’s performance. They are the important factors in optimizing chitosan’s application in food packaging [64,65,66,67]. So, incorporating chitosan into food packaging materials effectively inhibits microbial growth. It also meets the increasing consumer demand for natural and environmentally friendly additives in food packaging materials.

### 3.3. Synergistic Effects of Chitosan and Cellulose Blends in Antibacterial Activity

The synergistic effects of chitosan and cellulose in blends are pivotal in enhancing the functional properties of food packaging materials [13,15]. Chitosan has a cationic nature. When blended with cellulose, these two biopolymers complement each other, improving the overall performance of this kind of material [69]. The synergy between chitosan and cellulose arises from their properties to combine strong antimicrobial action with structural integrity and biodegradability.

**Figure 4:**
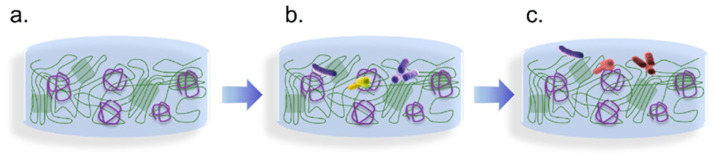
A schematic of chitosan and cellulose in blends interacting with microbials. (**a**–**c**) The antibacterial process of chitosan and cellulose in blends. (Purple thread represents chitosan, green thread represents cellulose, purple and yellow cylinders represent active bacteria, and reddish-brown cylinders represent dead bacteria.)

One significant aspect of this synergy is the improved antimicrobial efficacy observed when chitosan is incorporated into cellulose matrices. The presence of cellulose not only serves as a carrier for chitosan but also facilitates the controlled release of antimicrobial agents. This slow-release mechanism prolongs the antimicrobial effect, allowing for sustained inhibition of microbial growth over time [59,60,61,62,63,64]. The cellulose blend enhances the solubility and bioavailability of chitosan’s active components. So, it increases the contact between the antimicrobial agents and microbials. The result is particularly advantageous for food preservation. The schematic of chitosan and cellulose in blends interacting with microbials is displayed in Figure 4.

In addition, the mechanical and barrier properties are significantly improved through blending. Cellulose fibers provide strength and a higher degree of flexibility. It makes the resulting material more resilient for food packaging applications. Chitosan incorporation in blends enhances the tensile strength of the blend. It is essential for maintaining the freshness of food products [70]. The packaging materials with this combination can adapt to different types of food.

### 3.4. Antioxidant Properties of Chitosan–Cellulose Blended Films

Antioxidant properties also play a crucial role in food packaging materials. Oxidation can lead to the degradation of food quality, resulting in the loss of flavor, color, and nutritional value. Antibacterial chitosan–cellulose blended films in food packaging have garnered significant attention. Researchers have developed innovative packaging solutions by incorporating chitosan [13]. They help food to mitigate oxidative stress caused by environmental factors such as light, heat, and oxygen exposure [71]. These biopolymer blends inhibit microbial growth and provide a barrier against the food’s oxidative reactions.

Reactive oxygen species (ROS) refer to molecules, atoms, or ions that contain unpaired electrons. Three significant types of reactive oxygen species are superoxide anion radicals, hydroxyl radicals, and alkyl radicals. These free radicals exhibit high reactivity and are associated with over 100 diseases [72,73]. Moreover, reactive oxygen species can accelerate the aging and spoilage of fruits, vegetables, and fruit juices during storage, negatively impacting their quality and taste [74]. Therefore, researching and developing substances with antioxidant properties is significant. These substances can help mitigate the damaging effects of ROS, preserving the quality of food products.

Experimental studies have demonstrated that chitosan can scavenge free radicals and protect the body from oxidative damage [75]. Research indicates that low-molecular-weight chitosan has superior antioxidant properties compared with high-molecular-weight chitosan [76]. However, as molecular weight increases, the antioxidant capacity may decline. A similar trend has been observed in studies involving DPPH (1,1-diphenyl-2-picrylhydrazyl) radical scavenging [75,77].

**Figure 5:**
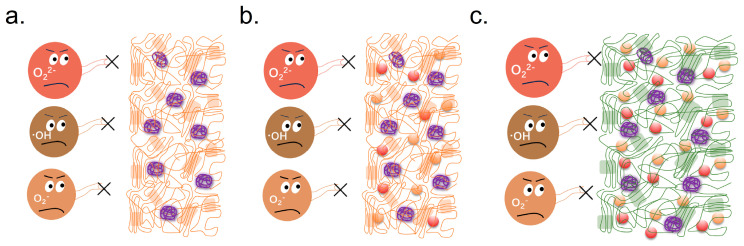
A schematic of different chitosan materials for free radical scavenging. (**a**) Chitosan and polymer film materials. (**b**) Modified chitosan and polymer composite film materials. (**c**) Modified chitosan and cellulose composite film materials. (Purple thread represents chitosan, green thread represents cellulose, orange thread represents other polymers.)

Microorganisms such as bacteria, molds, and yeasts significantly contribute to the spoilage of fruits, vegetables, meat, and seafood, leading to a reduced shelf life for these products [32]. The application of chitosan in food preservation can be classified into three categories: (1) the use of unmodified chitosan, as illustrated in Figure 5a; (2) modified chitosan; and (3) composite chitosan formulations. Unmodified chitosan possesses natural antimicrobial properties, effectively inhibiting the growth of spoilage-causing microorganisms [78].

Modified chitosan typically incorporates additional functional groups or nanoparticles to enhance its antimicrobial activity. The reason is that the functional groups or nanoparticles provide improved efficacy against a wider array of pathogens [79]. Composite chitosan, on the other hand, combines chitosan with other natural or synthetic materials to harness synergistic effects, resulting in even greater food preservation capabilities [80]. During the modification process of chitosan, its properties can be enhanced to improve its applicability, as shown in Figure 5b. For instance, chitosan’s poor water solubility and relatively weak antibacterial activity limit its practical use. Various modification techniques, such as acylation, esterification, and alkylation, can be employed to improve its solubility. Additionally, processes like sulfation, oxidation, reaction with heterocyclic compounds, and grafting can enhance its biological activity. Physical modifications can also be used to improve the mechanical properties of chitosan [80,81,82,83].

Modified chitosan and cellulose composite film materials can enhance antibacterial performance by adding preservatives or antibacterial agents to chitosan solutions. It has a better food preservation effect compared to using unmodified chitosan alone, as shown in Figure 5c. For example, antimicrobial agents such as sodium lactate, tea polyphenols, film-forming agents like glycerol, and antioxidants such as phytic acid can be incorporated to enhance their efficacy. Furthermore, adding other materials like silica, Tween, and nanomaterials can improve certain properties of chitosan [81]. Composite chitosan films demonstrate significantly better preservation performance than monolithic coatings. By leveraging these various additives, composite chitosan material maintains the desired functionalities and extends the shelf life of perishable products more effectively [82]. This adaptability makes composite chitosan an attractive option for food preservation and other related applications.

## 4. The Preparation of Chitosan–Cellulose Blends and Their Benefits and Drawbacks

### 4.1. The Preparation of Chitosan–Cellulose Blends

**Figure 6:**
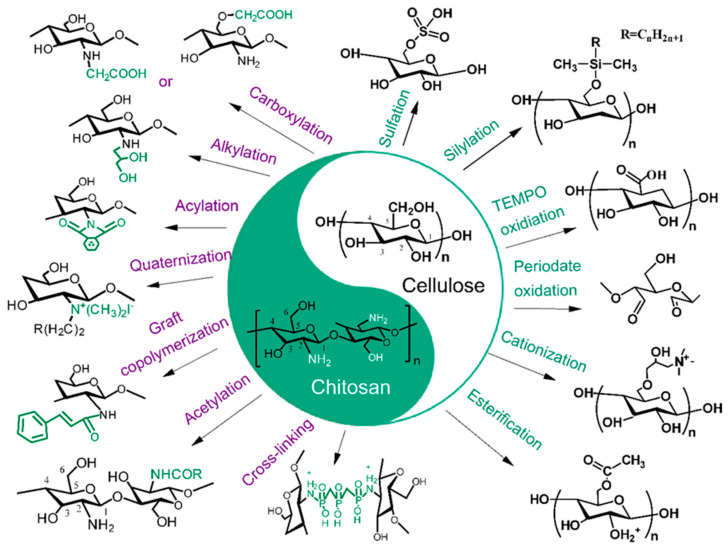
The different modifications of chitosan and cellulose in their blends for potential antibacterial food packaging.

The preparation process of chitosan–cellulose blends involves several methods. It focuses on the dissolution of biopolymers and subsequent mixing to create a homogeneous material. One common approach begins with the dissolution of chitosan in an acidic solvent, such as acetic acid. Acetic acid is beneficial for dissolving polymers while maintaining their inherent properties [13]. When components are effectively dissolved, they can be mixed in varying ratios. The process can help people to explore the optimal blend composition for the desired functionality. This blending process may also include the addition of plasticizers, such as glycerol, to enhance flexibility and processing characteristics. Different modified chitosan and cellulose can further enrich their applications. An illustration of chitosan and cellulose modifications in their blends for potential antibacterial food packaging is shown in Figure 6. For different modification studies, it is to optimize their performance in application fields such as food, sewage treatment, additives, etc., to meet practical application needs.

The significance and necessity of antimicrobial modifications in food packaging materials are multifaceted, primarily concerning food safety and sustainability. Antimicrobial packaging can inhibit the growth of bacteria, molds, and yeasts, thereby reducing the risk of foodborne pathogens, extending the shelf life of products, and minimizing food waste. These technologies cater to growing health awareness among consumers, differentiate products in the market, and assist companies in complying with food safety regulations, thereby enhancing consumer trust. Additionally, as the globalization of food progresses, food safety issues during long-distance transportation have become increasingly prominent, and antimicrobial packaging offers effective solutions to address these emerging challenges. In summary, antimicrobial food packaging not only helps improve product safety and freshness but also contributes to sustainable development, enhancing the integrity of the food supply chain. Based on these considerations, we explore the applications of chitosan and cellulose in green, renewable food packaging materials and the advantages and disadvantages brought about by their modifications, with the aim of providing a reference for their broader research and application.

For ease of understanding, we have conducted a comparative analysis of their different modification methods. Carboxymethyl chitosan is obtained after the carboxylation modification of chitosan. Compared with unmodified chitosan, its water solubility has been improved. In the chitosan molecule, the amino group at the C2 position and the hydroxyl groups at the C3 and C6 positions can be carboxylates. However, carboxylation of the hydroxyl group at the C3 position faces steric hindrance, making it less reactive, whereas carboxylation at the C2 amino and C6 hydroxyl groups is more common [83]. Alkylation is effective in food preservation because higher degrees of substitution and the presence of longer alkyl graft chains enhance coagulation-promoting effects. This leads to the improved stability and shelf life of food products [84,85]. The acylation modification of chitosan involves the reaction of its amino and hydroxyl groups with organic acyl chlorides or anhydrides. It produces O-acylated and N-acylated forms, increasing solubility and altering physicochemical properties according to reaction conditions. Research has found that the degree of acetylation significantly affects the properties of chitosan. Further research is crucial for fully understanding the behavior of modified chitosan and cellulose in food preservation applications. They include molecular interaction studies measured using surface force devices [75].

The quaternization reaction of chitosan is completed at the amino group on the C2 position, and it is typically carried out in two main ways. One is the direct quaternization of chitosan using alkyl halides. The other is grafting small molecules containing quaternary ammonium salt groups onto chitosan to achieve quaternized chitosan [86]. Chemical modifications of natural polysaccharides enable diverse applications. And the development of tailored hybrid substances is completed through graft copolymerization methods. By grafting synthetic monomers onto natural chitosan, desired properties can be enhanced, thereby expanding the range of potential applications by incorporating various side chains [87]. In chitosan molecules, the amino and hydroxyl groups can react with cross-linking agents such as polyaldehydes, polycarboxylic acids, epichlorohydrin, episulfides, polycarboxylic anhydrides, and polyethers. These reactions increase intermolecular cross-linking, resulting in more stable chitosan derivatives, although their solubility decreases [88].

Negatively charged sulfate polysaccharides play a crucial role in their biological activity, promoting both specific and non-specific interactions with positively charged proteins. Additionally, the antibacterial properties of sulfate cellulose, combined with its promising biomedical applications, make it one of the most valuable cellulose derivatives on the market [89]. In recent years, research into silylation technology has deepened to further enhance the performance of cellulose. Silylation involves incorporating multifunctional silane compounds into cellulose fibers or nanocrystals, endowing them with unique characteristics based on specific functional groups within glucose units. This process not only increases the crystallinity and tensile strength of cellulose materials but also improves their functional capabilities, such as water vapor barrier properties and thermal resistance, making them more suitable for various applications [90]. Meanwhile, since 2017, the application range of TEMPO-oxidized cellulose nanofibers (TOCNFs) has also expanded significantly. This type of material has a carboxylate content of approximately 1.7 mmol/g and is increasingly favored in various fields due to its uniform width, excellent crystallinity, and high tensile strength. TOCNF is widely used in adhesives, hydrogels, membranes, and medical applications [91]. The enhancement of these properties is closely related to advancements in sulfation and silylation technologies, providing new opportunities for the development of cellulose-based materials in the biomedical field. Therefore, the processing techniques of sulfation, silylation, and TEMPO oxidation together drive the diversified applications of cellulose-based materials, opening new avenues for future research and development.

Periodate oxidation has proven effective for isolating nanocellulose. Particularly, when the oxidation degree is carefully controlled, it leads to advances in nanofibrillation techniques. This method has gained attention for producing dialdehyde cellulose nanocellulose. It also shows great potential for further functionalization and diverse applications in nanocellulosic material development [92,93]. Cellulose cationization was achieved through direct or indirect graft polymerizations. The graft methods were then evaluated for their effects on functionalization degree, thermal stability, crystallinity, and antiviral activity of cellulose. Indirect cationization yielded the highest polymer grafting, enhancing particle size and thermal stability. And the antiviral efficacy depended on the specific structure of functional groups and surface charge density. These changes demonstrate their potential for applications in textiles and packaging [94]. Cellulose esters are promising bio-based materials with potential applications in coatings, films, and plastics. These applications are based on their internal plasticization properties. Various esterification methods can synthesize cellulose esters with different side chain lengths. The acyl chloride method proved to be the most effective under homogeneous conditions [95,96].

### 4.2. The Benefits and Drawbacks of Different Modifications of Chitosan–Cellulose Blends

**Figure 7:**
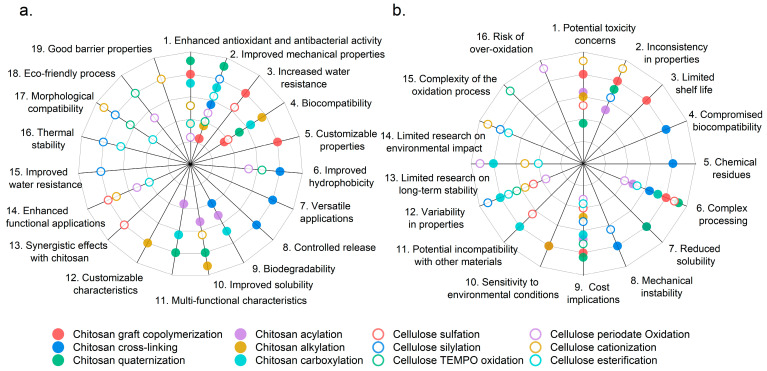
The benefits (**a**) and drawbacks (**b**) of different modifications of chitosan and cellulose in their blends for potential antibacterial food packaging.

To facilitate the analysis and understanding of the benefits and drawbacks of different modifications of chitosan and cellulose in their blends for potential antibacterial food packaging, we present these aspects in the form of radial bar figures in Figure 7. Solid circles of different colors represent various modified chitosan samples, while hollow circles of different colors represent various modified cellulose samples. In the left figure (Figure 7a), the advantages of modified chitosan and cellulose can be directly observed. Similarly, in the right figure (Figure 7b), the drawbacks of the modified chitosan and cellulose are clearly represented. Some modified chitosan samples, such as chitosan cross-linking (indicated by the blue solid circle), exhibit five advantages (Figure 7a), while the corresponding drawbacks associated with the modifications amount to four (Figure 7b). This figure can also be a reference for future research on modified chitosan and cellulose blends in different application scenarios.

Chitosan graft copolymerization in Figure 7 offers significant advantages, including enhanced antibacterial properties, improved mechanical strength, increased water resistance, and customizable characteristics, making it a valuable choice for food packaging applications. However, it poses challenges such as higher costs, complex manufacturing processes, potential toxicity concerns, inconsistent material properties, and limited shelf life [56,97,98]. Chitosan cross-linking enhances mechanical properties, hydrophobicity, and versatility for applications in packaging and biomedicine. Meanwhile, it retains biodegradability and facilitates controlled release of active ingredients. However, it may compromise biocompatibility, introduce chemical residues, complicate processing, reduce solubility, and create mechanical instability over time [99,100,101]. Chitosan quaternization significantly enhances antibacterial activity, solubility, biocompatibility, mechanical properties, and multifunctional characteristics, making it suitable for various food packaging applications. Nevertheless, it involves higher costs, a complex manufacturing process, potential toxicity concerns, and inconsistencies in material properties, and it may exhibit limited stability over time [45,102,103]. Chitosan acylation enhances antibacterial properties, allows for targeted functionalization, improves mechanical and barrier properties, offers customizable solubility, and maintains biodegradability, making it suitable for food packaging applications. Nonetheless, the process can be complex and costly, potentially leading to inconsistencies in material properties, raising toxicity concerns depending on the acylating agents, and exhibiting sensitivity to environmental conditions [38,79,104]. Chitosan alkylation enhances antibacterial activity, solubility, mechanical properties, and biocompatibility while allowing for customizable characteristics tailored for specific food packaging applications. On the other hand, it involves complex processing, higher costs, potential inconsistencies in material properties, toxicity concerns regarding alkylating agents, and sensitivity to environmental conditions [36,75,105,106]. Chitosan carboxylation enhances antibacterial activity, solubility, and mechanical properties, and it allows for tailored functionalities, making it suitable for eco-friendly food packaging applications. However, the process is complex, may increase production costs, can lead to material compatibility issues, exhibits variability in different properties, and has limited research on long-term stability under various environmental conditions [107,108,109].

Cellulose sulfation enhances antibacterial properties, solubility, and functional applications while allowing for synergistic effects with chitosan, making it advantageous for food packaging. Nonetheless, the process is complex, may increase production costs, can alter mechanical properties, leads to variability in material consistency, and has limited research on its long-term environmental impact [110,111,112,113]. Cellulose sialylations enhance mechanical properties, water resistance, thermal stability, and compatibility with chitosan, making it effective for durable food packaging applications. On the other hand, the process is complex and costly, raises potential toxicity concerns, reduces biodegradability, and may lead to variability and incompatibility with other materials [112,114,115]. Cellulose TEMPO oxidation enhances mechanical properties, hydrophilicity, antibacterial activity, and compatibility with chitosan, while also being an eco-friendly modification process. The disadvantage is that this method is complex and costly, which may lead to performance changes; has limited long-term stability data; and may affect biodegradability [91,116,117,118]. Cellulose periodate oxidation enhances functionalization, antibacterial properties, mechanical strength, and modifiable hydrophilicity, making it compatible with eco-friendly packaging materials. Nonetheless, the process can be complex and costly, with risks of over-oxidation, variability in product quality, and limited long-term stability data [92,93]. Cellulose cationization improves antibacterial properties, water solubility, compatibility with chitosan, and barrier performance, making it beneficial for packaging applications. However, the complexity of the cationization process, potential cost implications, changes in properties, and limited long-term stability data may hinder its commercial viability [119,120,121]. Cellulose esterification enhances barrier properties, mechanical strength, and compatibility with chitosan while allowing for tailored functional properties and improved antioxidant and antibacterial activity, making it beneficial for food packaging applications. Nevertheless, the complexity of the process, potential cost increases, variability in product quality, limited long-term stability data, and concerns about biodegradability limit its commercial applicability [122,123,124].

In the above content, we analyzed the advantages and disadvantages of different modification methods for chitosan and cellulose through Figure 6. Modified chitosan and cellulose materials exhibit excellent performance in enhancing antioxidant and antibacterial activity, improving mechanical properties, and biocompatibility. These materials may not be satisfactory in terms of complex processing, potential toxicity issues, variability in performance, and cost impact, but these are also important research areas for future development.

## 5. Future Directions for Chitosan–Cellulose Blends

The future of chitosan–cellulose blends holds remarkable potential for innovation. These improving aspects include enhancing their properties to address the growing demands for effective, sustainable, and multifunctional packaging solutions.

### 5.1. Integration of Nanotechnology

One of the most promising areas for innovation is the integration of nanotechnology into chitosan–cellulose blends. Incorporating nanoparticles, such as silver, titanium dioxide, or even quantum dot materials, can significantly enhance the packaging material’s antibacterial properties and mechanical strength [125]. M.M. Abutalib synthesized silver (Ag) nanoparticles with an average crystal size of 20 nm using water extract of fresh quinoa leaves, combined with 15 nm TiO_2_ nanoparticles, and added Ag and TiO2 nanoparticles to the polymer blend system. The activity indices (%) of the antibacterial activity of the sample mixture + (0.3%) Ag + (0.8%) TiO_2_ against Escherichia coli, Staphylococcus aureus, Candida albicans, and Aspergillus niger were 32%, 45.8%, 77.8%, and 92%, respectively. This provides new insights into the applicability of nanocomposites in food packaging applications [126]. Recently, quantum dots of MXene have been integrated into thermoplastic chitosan through wet chemical blending method, resulting in nanocomposite films with excellent UV resistance (>90%), antioxidant activity (>78%), and good flexibility at −30 °C. This makes it a promising alternative material for sustainable and high-performance food packaging solutions [127].

These nanoparticles can provide a larger surface area for antimicrobial activity and improved interaction with the food products. Furthermore, nanomaterials can bolster the barrier properties against gases and liquids, extending shelf life while maintaining freshness. Advances in nanotechnology also allow for the precise control of the size, shape, and distribution of particles within the polymer matrix, leading to the optimization of overall performance.

### 5.2. Functionalization and Surface Modification

Functionalization techniques that modify the chemical structure of chitosan and cellulose can yield significant improvements in their properties. Methods such as graft copolymerization or blending with other biopolymers can create new materials with enhanced properties, such as improved adhesion, water resistance, and flexibility. For example, introducing functional groups that react favorably with food products can improve the seal ability of packaging, thereby enhancing its protective characteristics. The hydrophobicity of polyvinyl alcohol (PVA) and chitosan (CS) composite films is stronger than that of PVA films. This composite film has higher mechanical properties, with a Young’s modulus, tensile strength, and elongation at break of 344.99 MPa, 39.12 MPa, and 507.09%, respectively [128]. Hydrophobic microcrystalline cellulose ester was prepared using microcrystalline cellulose and long-chain stearic acid. The above sample was applied onto the surface of sugarcane bagasse fiber paper to form a continuous hydrophobic film. This material exhibited good water repellency and oxygen blocking activity. The coated material samples also showed excellent dimensional stability, good wet tensile strength of 16 MPa, and good antibacterial performance in water [129]. Research has shown that modified composite films can extend the shelf life of antibacterial packaging. Surface modifications, such as creating hydrophobic or hydrophilic surfaces, can help tailor the blends for specific applications, allowing for better compatibility with various types of food items and environments.

### 5.3. Smart Packaging Solutions

The emergence of smart packaging is set to revolutionize food packaging made from chitosan–cellulose blends. Integrating sensors that monitor temperature, humidity, and the presence of spoilage indicators can provide real-time data on food quality and safety.

Zhiming Guo et al. recently developed a detector for collecting volatile gases from apples infected with acute anthrax, Botrytis cinerea, and Botrytis cinerea using deep learning and variable selection algorithms. This device can transmit data in real-time and monitor remotely, achieving effective analysis and grading alerts for apple spoilage. It is expected that when combined with other different food packaging materials, it can achieve intelligent and powerful real-time monitoring and improve food quality. In addition, this study applied a collaborative interval gated cyclic unit model to construct an optimal warning model for multi-environmental factor detection [130]. In addition, color-changing indicators can alert consumers to potential spoilage, enhancing food safety and reducing waste. Vânia Gomes researched intelligent labels for freshness monitoring in food packaging. In summary, 0.2% (*w*/*w*) pyranoyl pigment and 30% (*w*/*w*) glycerol were added to a cellulose solution. Thin films with different pH response properties (pH 4 to 8) were prepared by the solvent casting method. In the preservation of fish and meat, freshness monitoring of fish samples can be carried out. As the fish meat rots, the yellow label begins to turn purple, effectively helping people detect the beginning of the fish meat spoilage process [131]. These innovations will benefit consumers and empower manufacturers to track product conditions better throughout the supply chain. Smart packaging can help people store food under optimal conditions.

### 5.4. Advanced Processing Techniques

The advancement of processing techniques will play a crucial role in the future of chitosan–cellulose blends. Techniques such as 3D printing and electrospinning can create structures with highly tailored properties, enabling the design of innovative packaging solutions. For example, electrospinning can produce ultrafine fibers that enhance the surface area available for antibacterial action [132]. Meanwhile, 3D printing allows for customizable shapes and designs that improve the packaging’s protective capabilities and consumer appeal. Three-dimensional printing technology is expected to be applied in food production for different populations with varying demands by adding different food ingredients and additives. The demands mainly include customized printing of food, personalized nutrition, and food packaging applications. 

In addition to meeting the requirements of color, size, and design, additive manufacturing technology through 3D printing can also reduce waste of packaging materials. On the other hand, customized food packaging can be achieved through 3D printing to explore the optimal application process and conditions of additive manufacturing in food packaging. Three-dimensional printing technology is also used to develop machine parts for packaging production lines in the food packaging process. These include picking and placing robots. This innovation reduces the time and resources required for outsourcing design and manufacturing operations [133]. In addition, by combining the characteristics of 3D printing with thermal environment temperature sensing systems in the production process of food packaging, research can be conducted to improve the thermal performance and environmental adaptability of food packaging [134]. So, 3D printing developments in bio-composite processing will facilitate large-scale manufacturing and integration with existing packaging technologies.

## 6. Conclusions

Chitosan and cellulose are gaining attention in the food industry due to their eco-friendly characteristics and broad application prospects. Both biopolymers are derived from renewable sources, making them sustainable alternatives to conventional plastic materials, a significant consumer concern today. One of the most promising applications of chitosan and cellulose blends lies in food packaging. Chitosan possesses notable antimicrobial properties that can inhibit the growth of bacteria and fungi, while cellulose provides essential structural integrity, mechanical strength, and flexibility. This combination enhances food preservation and contributes to reducing plastic waste, aligning with global sustainability goals.

Moreover, the development of chitosan and cellulose blends involves various preparation methods that can be tailored to enhance their properties. Research is progressing toward customizing these blends for specific food types and applications, increasing their effectiveness in preserving food quality and safety. As consumer preferences shift towards safer and more sustainable packaging options, innovation in using chitosan and cellulose blends is poised to play a crucial role in the future of the food packaging industry. This paper aims to encourage further research on the structure and properties of these blends following various modifications, highlighting their potential to revolutionize food packaging and contribute to environmental sustainability.

## Figures and Tables

**Table 1:** The application of different antibacterial films of chitosan and cellulose blends.

	Evaluation Criteria		
Materials Components	Preparation Approaches	Applications	References
1. Alkylation of chitosan	Chitosan undergoes N-alkylation through the formation of Schiff bases	Antibacterial, food, and pharmaceutical	[36]
2. Cellulose alkylation	Alkylation of micro-fibrillated cellulose	Food preservation	[37]
3. Chitosan acylation	Chitosan acylation, biocatalytic enzyme activity	Food preservation	[38]
4. Acetylated cellulose	Reacetylation method; banana pseudostem cellulose was extracted and acetylated to prepare	Beef preservation; antimicrobial against Staphylococcus aureus and Escherichia coli	[34,35]
5. Hydroxychitosan	Hydroxyl substitution of flavonols on chitosan	The preservation of fatty and water-based meats	[39]
6. Imine-based chitosan/quaternized chitosan-based nanofibers	Ammoniated chitosan, vanillin, and polyethylene oxide electrospun into fiber materials	Antibacterial activity of Escherichia coli, Staphylococcus aureus, and Candida albicans	[40]
7. Shellac quaternized chitosan nanoparticles	Quercetin-loaded shellac quaternized chitosan nanoparticles	Cosmetics, pharmaceuticals, and food preservation	[41]
8. Quaternary ammonium chitosan, cellulose	Deacetylated quaternized chitosan and its use as cellulose nanofiber-based film	Extend the raw salmon’s shelf life	[42]
9. Amphiphilic quaternized chitosan derivatives	Amphiphilic quaternized chitosan derivatives	Antibacterial experiment of Streptococcus mutans, the pathogen of dental caries	[43]
10. Polyvinyl alcohol/quaternized cellulose	Blending of quaternized cellulose with polyvinyl alcohol matrix	Antibacterial experiments on Gram-positive (Staphylococcus aureus) and Gram-negative (Escherichia coli) bacteria	[44]
11. derivatives of cellulose, chitin and chitosan	Introduced multifunctional groups	Fruits, antibacterial field, fuel cell, drug delivery, immunotherapy, etc.	[45]
12. Cellulose nanofibers, chitosan/modified cellulose	Physical mixing	Beefcake food preservation	[46]
13. Cellulose, gelatin, starch chitosan nanocomposite film	Single-layered films through film casting technique	Meat preservation	[47]
14. Microfibrillated cellulose, chitosan and polypyrrole	Coasting method	Cherry tomato preservation	[48]
15. Cellulose nanocrystals, polyvinyl alcohol, chitosan nanoparticle	The solvent casting method	The packaging of fresh fruits	[49]
16. Carboxymethyl cellulose, chitosan-based carbon quantum dots	Carboxymethyl cellulose-based functional film integrated with chitosan-based carbon quantum dots.	Lemon fruit preservation	[50]
17. Chitosan, lignin-containing cellulose nanofibrils bio-composite	Combining hydrothermal pretreatment, mechanical fibrosis, and casting.	Green food packaging	[51]
18. Hydrophobic-modified cellulose nanofibrils, chitosan, zein coating	Multi-coating method	Meat packaging	[52]
19. chitosan/cellulose acetate hybrid nanostructure, Ziziphora clinopodioides essential oils.	Ion gel, electric spray, and electrospinning process	Fresh beef preservation	[53]
20. Cellulose and chitosan and volatile antibacterial benzyl isothiocyanate.	The layer-by-layer self-assembly approach	Chicken preservation	[54]
21. Aluminum chloride, chitosan, cellulose	Ternary composite approach	Gram microbiota experiment	[55]
22. Chitosan, bacterial cellulose, ε—polylysine	Casting method	Tilapia preservation	[56]
23. Corn alcohol soluble protein, cinnamaldehyde, chitosan, dialdehyde carboxymethyl cellulose	Loading and doping method	Strawberry preservation	[57]
24. Composed of cellulose, bentonite, and chitosan, Aspergillus Niger extract	Ternary composite approach	Sherbet berry preservation	[58]

## Data Availability

No new data were created or analyzed in this study. Data sharing does not apply to this article.
